# Supplementary material for: NIR and Reduction Dual-Sensitive Polymeric Prodrug Nanoparticles for Bioimaging and Combined Chemo-Phototherapy
Source: Polymers (Basel). 2022 Jan 11;14(2):287. doi: 10.3390/polym14020287 (PMC8779475; doi:10.3390/polym14020287)
Supplement: Supplementary file 1 [file polymers-14-00287-s001.zip › polymers-1506539-supplementary.pdf]

[1]Supporting Information

NIR/Reduction Dual-sensitive Polymeric Prodrug Nanoparticles for Bioimaging and Combined Chemo-phototheray

Shuying Li[1], Yanjuan Wu[1*], Xiukun Xue[1], Siyuan Liu[1]

S. Y. Li, X. K.Xue, Dr. Y. J. Wu, Dr. S. Y. Liu

Shandong Provincial Key Laboratory of Molecular Engineering, School of Chemistry and Chemical Engineering, Qilu University of Technology (Shandong Academy of Sciences), Jinan 250353, China;

E-mail: wuyanjuan5@qlu.edu.cn;

Synthesis of DHP

According to reports in the literature[38], cisplatin (1.0 g, 3.33 mmol) was added to a 50 mL flask, 10 mL ultrapure water and 10 ml hydrogen peroxide (H_2O_2, 30%) were added, and stirred for 12 h in the dark. After the filtration and washing with ultrapure water and ice-cold acetone several times, the obtained solid cis,cis,trans-$[Pt(NH_3)_2Cl_2(OH)_2]$ (DHP) was dried in vacuum to obtain 0.85g light yellow powder solid, the yield of DHP was 85%.

Synthesis of Pt(IV) prodrug HO-Pt-COOH

DHP (0.4g, 1.2mmol), succinic anhydride (0.12g, 1.2mmol), and DMSO (4ml) was added to the polymerization vial, with a light avoidance reaction at 30°C for 24h in the inert gas state [39]. After the reaction, the solvent was drained, dissolved with 2 mL methanol, then precipitated in ice-cold anhydrous diethyl ether, The obtained solid product was filtered and dried under vacuum, the product of cis,cis,trans-$[Pt(NH_3)_2Cl_2(OH)(O_2CCH_2CH_2CH_2CO_2H)]$(HO-Pt-COOH) was 0.45g (87%).

Synthesis of Cy

According to the literature reports [37], 20 mL anhydrous DMF and 20 mL

anhydrous DCM were added into a two-necked flask in an ice-water bath. Subsequently, 20 mL $POCl_3$ was slowly dropwised with a constant pressure dropping funnel, and then cyclohexanone (5 g, 51 mmol) was gradually pumped into the flask. Finally, the mixture was reacted for 7 hours at 55°C in the dark under N_2 atmosphere. After concentration and recrystallization, a yellow solid, 1-cyclohexene-1-carboxaldehyde was obtained **(I)**. 2,3,3-trimethylindolenine (6 g, 37.7 mmol), 2-bromoethanol and 80 mL of anhydrous acetonitrile were added to a single-necked flask, the reaction was carried out at 85°C in the dark under N_2 atmosphere for 48h. After rotary evaporation and concentration, The crude product was settled in frozen ethyl acetate and dried to obtain a pink solid, 1-(3-hydroxy ethyl)-2,3,3-trimethyl-3H-indol-1-ium**(II)**.

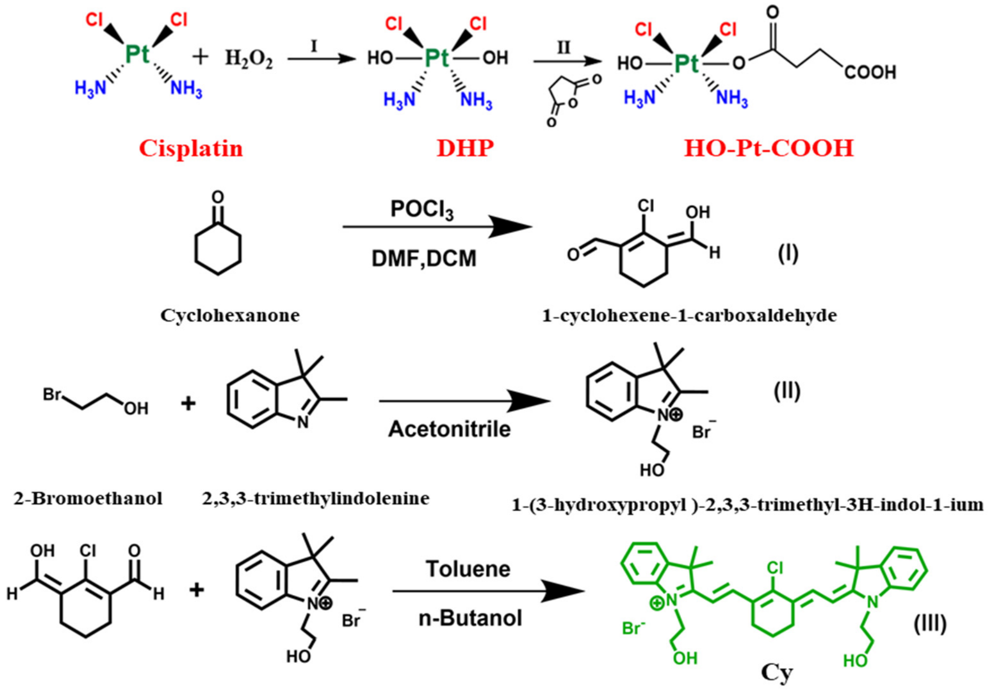

Scheme S1. Synthetic scheme of the DHP(I), HO-Pt-COOH(II) and Cy.

Dissolve 1-cyclohexene-1-carboxaldehyde (1.73 g, 10 mmol) and 1-(3-hydroxy ethyl)-2,3,3-trimethyl-3H-indol-1-ium (5.735 g, 20 mmol) in anhydrous n-butanol (70 mL) and toluene (30 mL), and react in N_2 atmosphere at 120°C in the dark for 12 h, the solvent was separated and collected by an oil-water separation device, and after cooling to room temperature, the crude product is separated and purified by a chromatographic column to obtain

1-(2-hydroxyethyl)-2-((E)-2-((E)-3-((E)-2-(1-(2-hydroxyethyl)-3,3-dimethylindolin-2-ylidene)ethylidene)-2-chlorocyclohex-1-en-1-ly)vinyl)-3,3-dimethyl-3H-indol-1-ium bromide (Cy)**(III)**, the Cy was 3.48g, the yield was 47%.

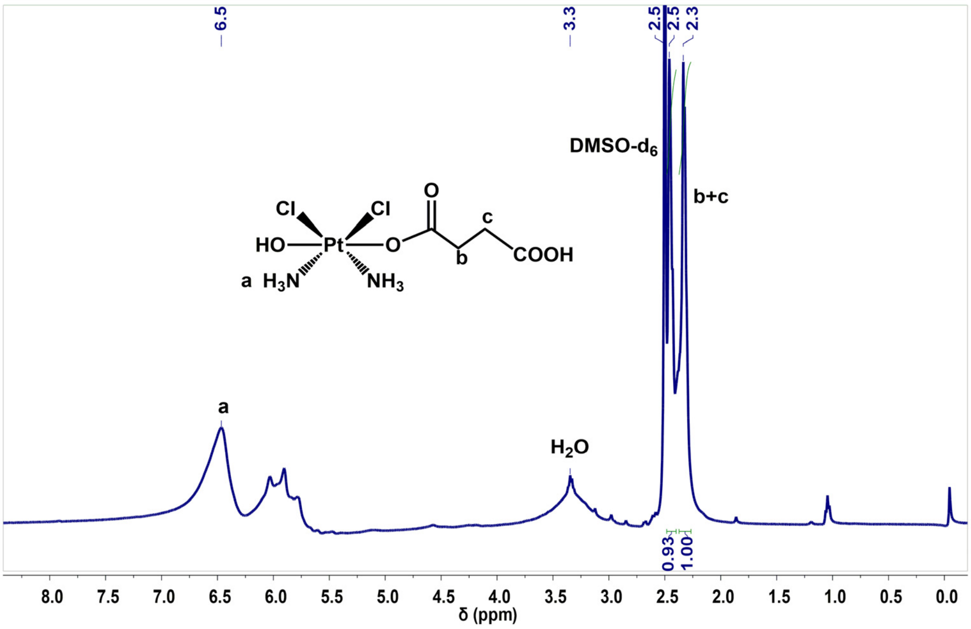

Figure. S1. ^{1}H NMR spectrum of HO-Pt-COOH in DMSO-d_6.

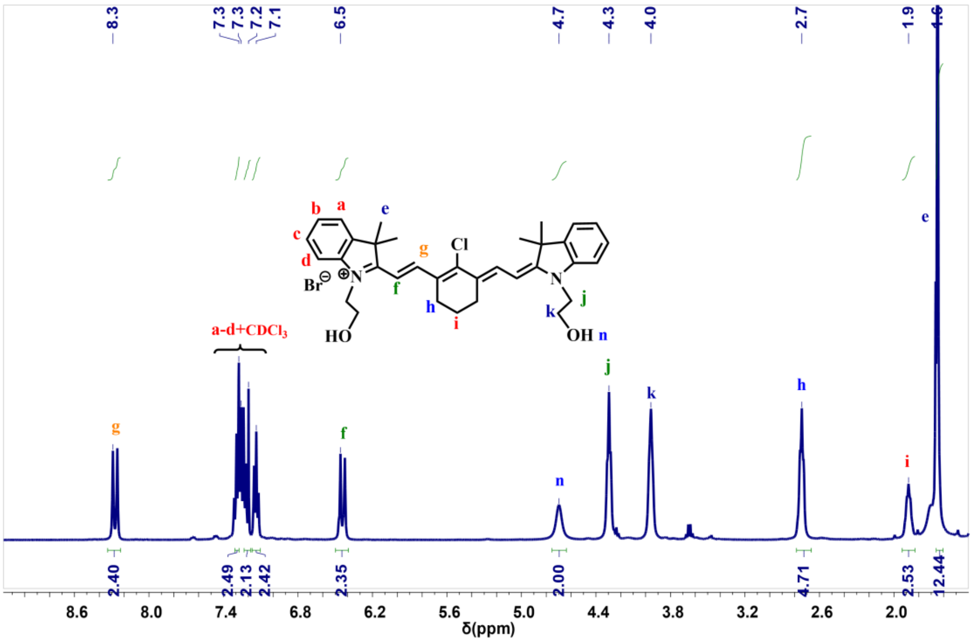

Figure. S2. ^{1}H NMR spectrum of Cy in $CDCl_3$.

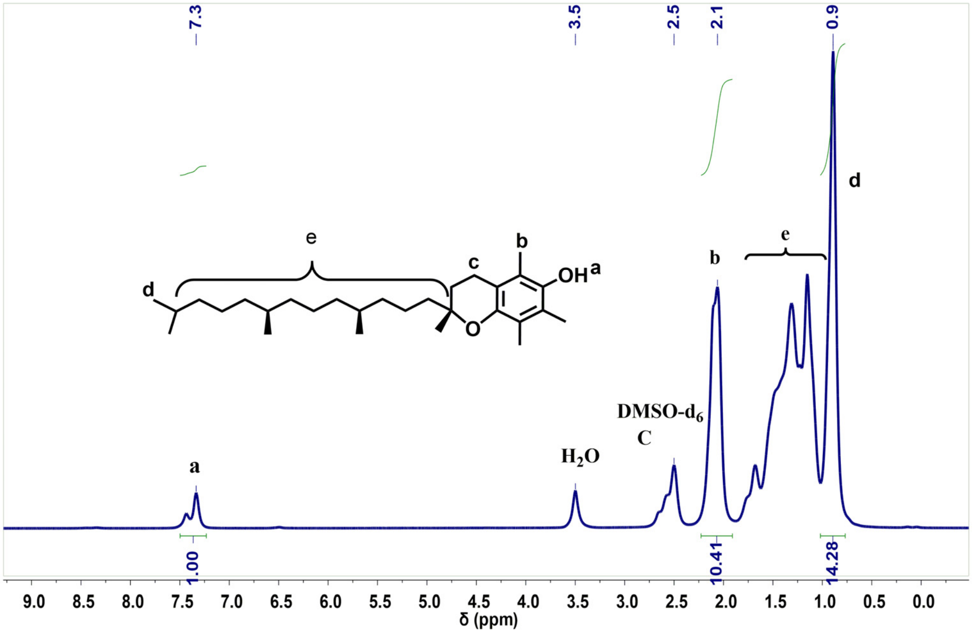

Figure S3. ^{1}H NMR spectrum of α-tochoferol in DMSO-d6.

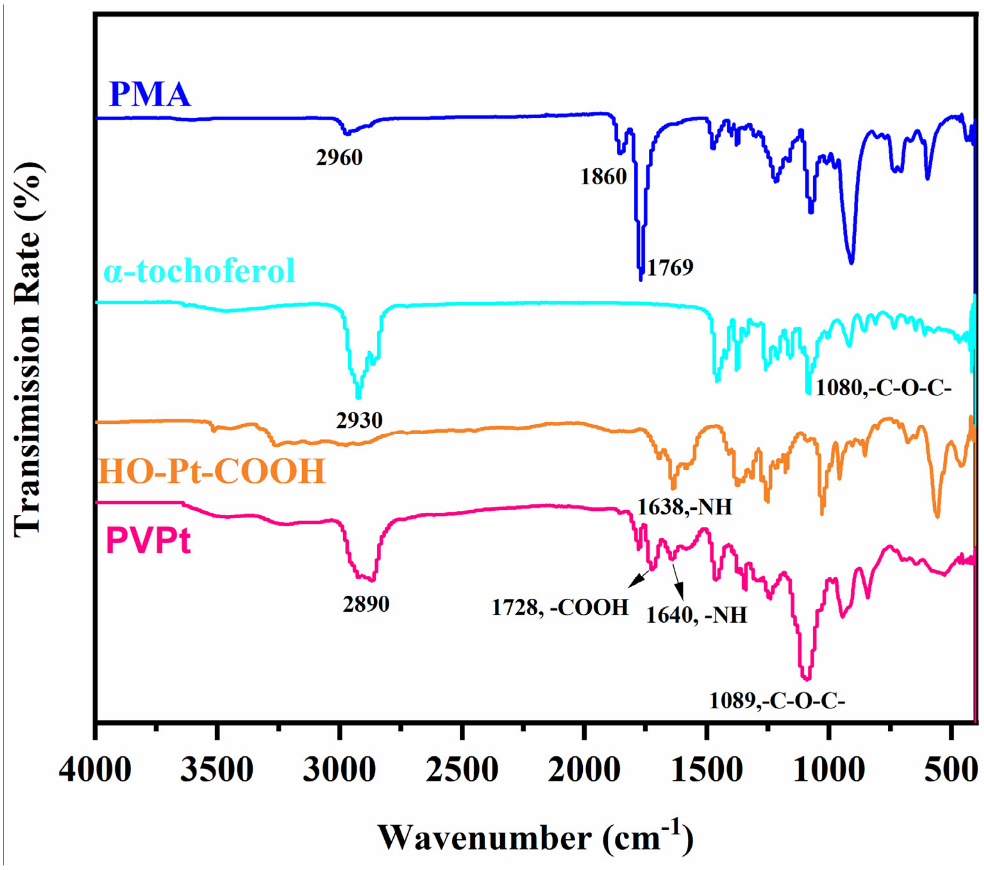

Figure S4. FT-IR spectra of PMA, α-tochoferol, HO-Pt-COOH and PVPt.

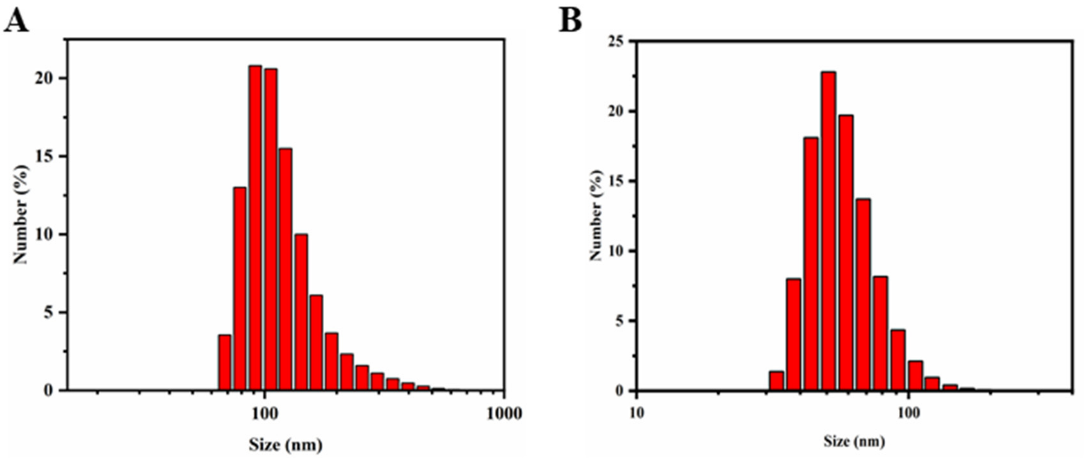

Figure S5. Hydrodynamic size of PVPt NPs (A) and PVPt@Cy NPs (B) after re-dispersion.

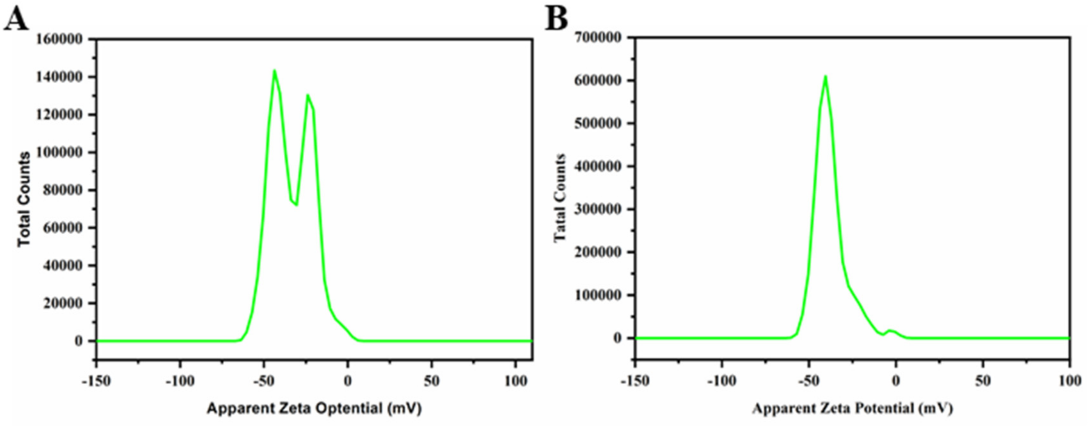

Figure S6. Zeta potential measurement of PVPt NPs(A) and PVPt@Cy NPs(B).

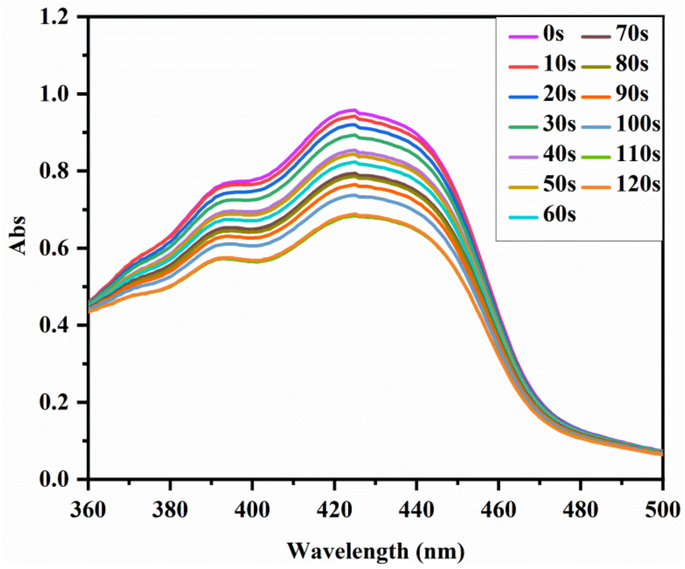

Figure S7. Absorption spectra of DPBF solution incubated with PVPt@Cy NPs in the dark (A) or under 808 nm irradiation (B) for different times.

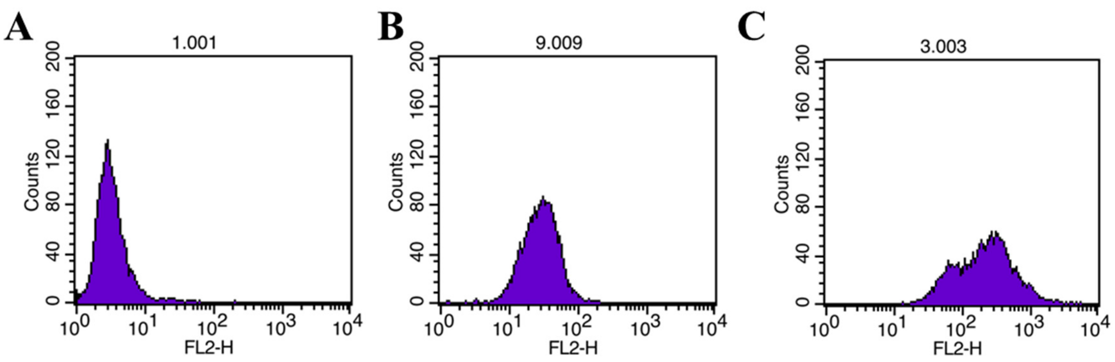

Figure S8. Flow cytometric histogram for quantitative comparison of the cellular uptake of control (A), PVPt@Cy NPs for 2 h (B), and PVPt@Cy NPs for 12 h (C).

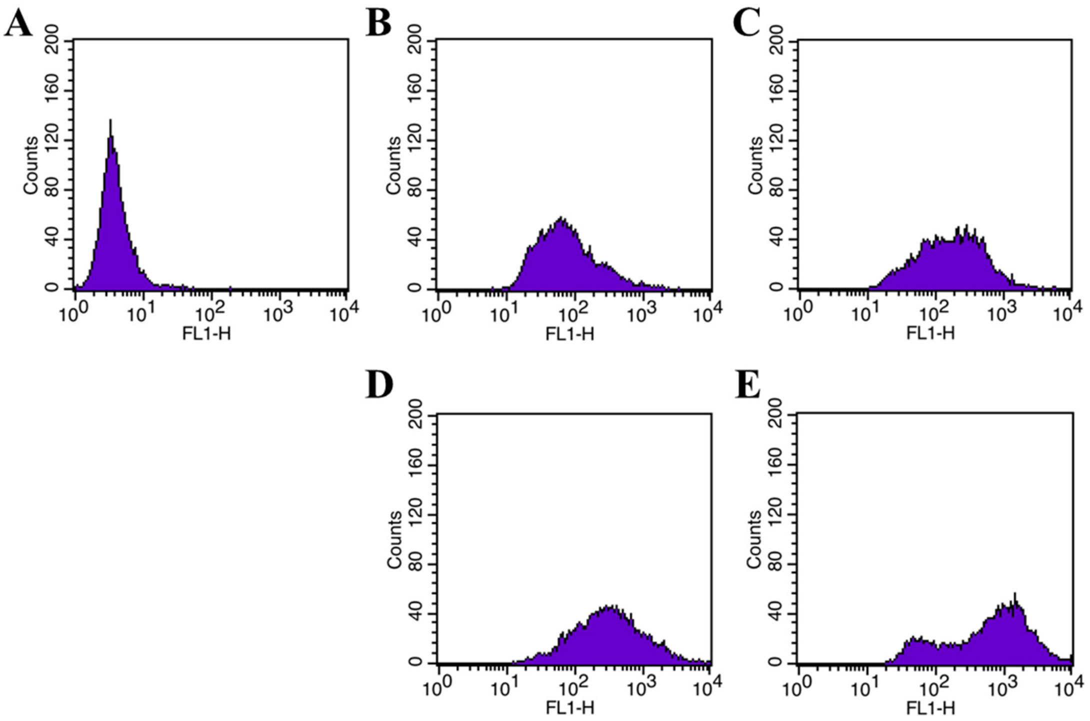

Figure S9. Flow cytometric histogram for quantitative comparison of the intracellular ROS generation of control (A), Cy in the dark (B), PVPt@Cy NPs in the dark (C), Cy with 808 nm irradiation (D), and PVPt@Cy NPs with 808 nm irradiation (E).
